# Supplementary material for: Disease Activity-Dependent Siglec-1 Expression on Monocyte Subsets of Patients with Idiopathic Inflammatory Myopathies
Source: Int J Mol Sci. 2025 May 21;26(10):4950. doi: 10.3390/ijms26104950 (PMC12112322; doi:10.3390/ijms26104950)
Supplement: Supplementary file 1 [file ijms-26-04950-s001.zip › ijms-3615331-supplementary.pdf]

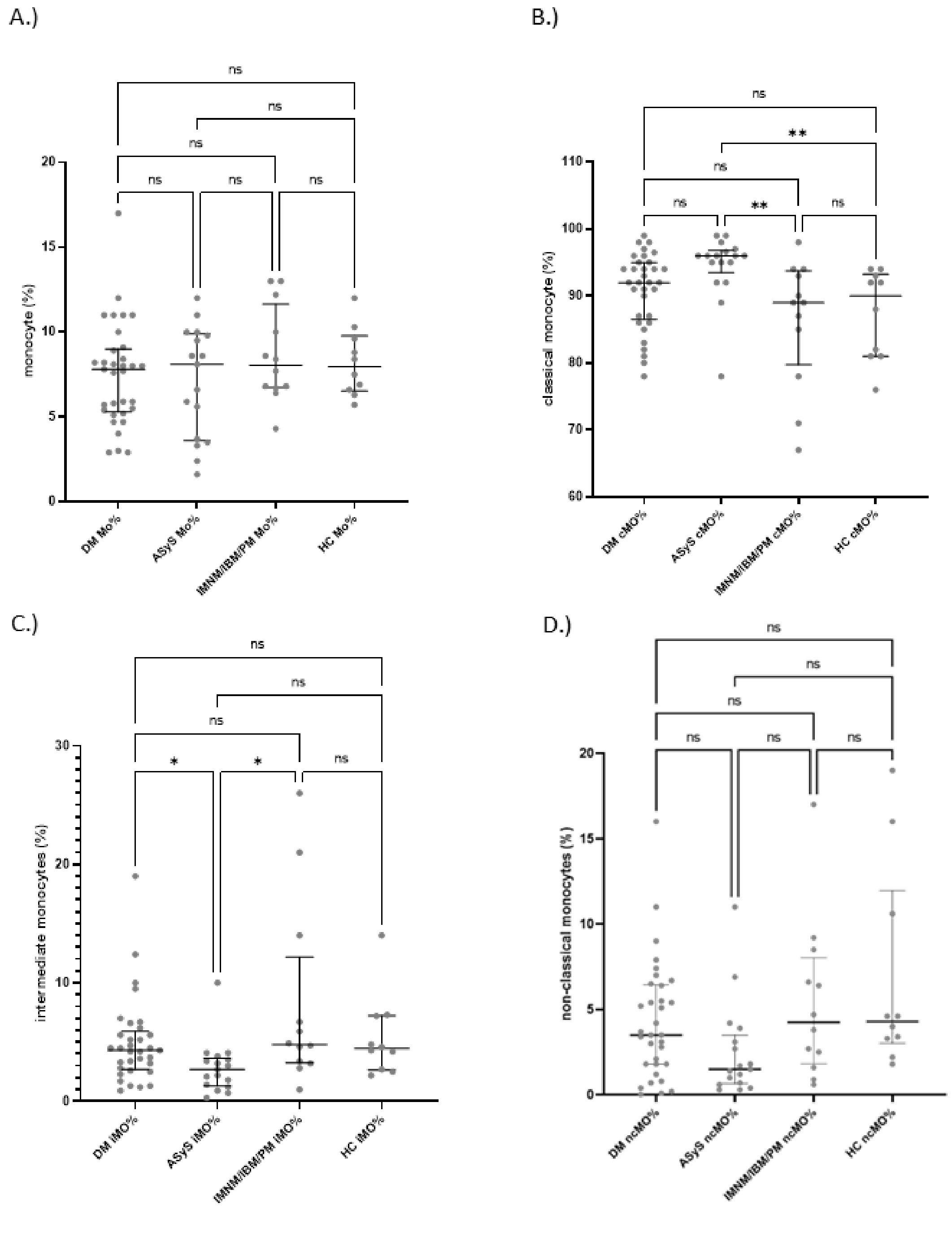

Figure S1. Comparison of proportions of monocytes (A.) and their subpopulations (B-D.) among patient groups. Examining the distribution of subpopulations across different patient groups, the proportion of classical monocytes was significantly elevated in the ASyS group compared to the DM and IMNM/IBM/PM groups, whereas the proportion of intermediate monocytes was significantly lower. No differences were observed in the proportion of non-classical monocytes among the patient groups.

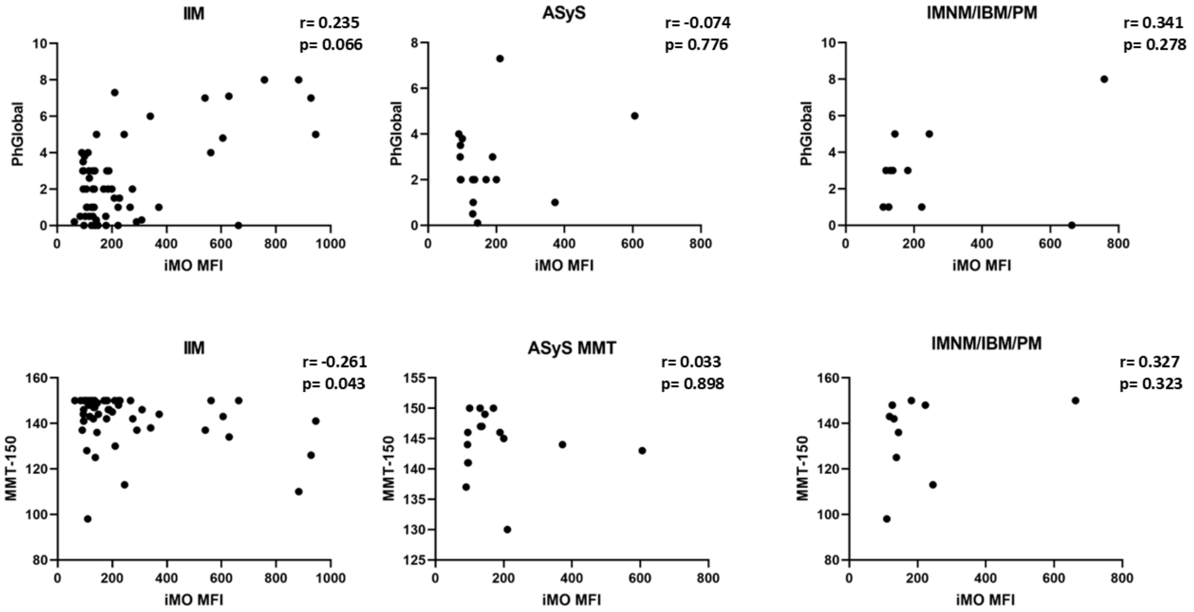

Figure S2. Correlation of Siglec-1 MFI values of intermediate monocytes with disease activity parameters, such as Physician Global Disease Activity (PhGlobal) and muscle force (MMT-150) in the whole group of myositis patients (IIM) and in subgroups other than dermatomyositis (ASyS, IMNM/IBM/PM).

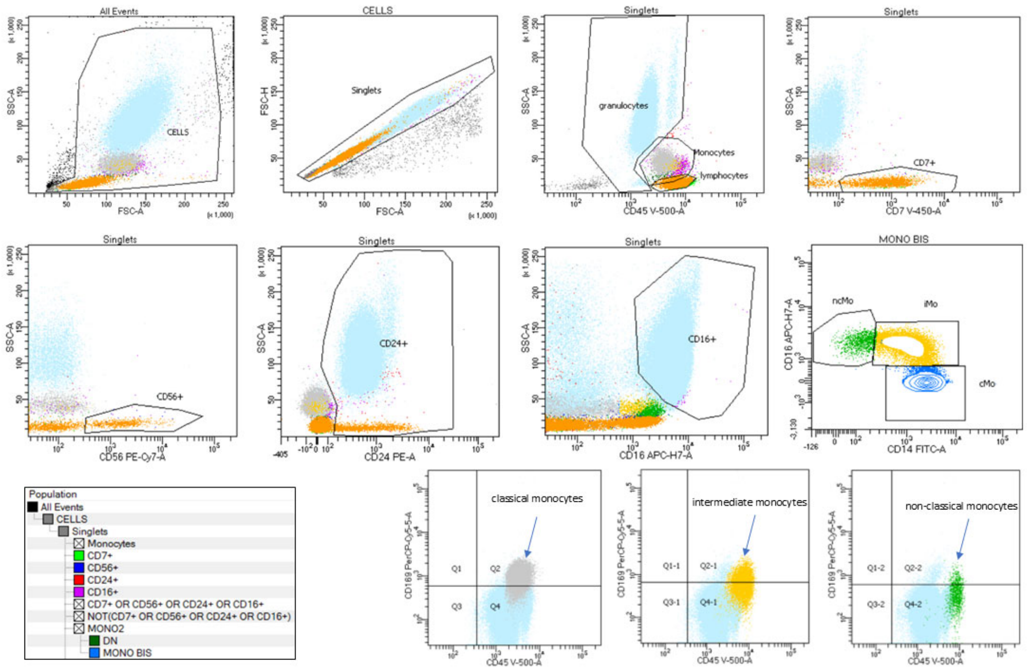

Figure S3. A gating strategy to identify specific cell types. Live cell assignment was based on SSC and FSC parameters. After identification of singlets capitation of major white blood cell types was carried out by using SSC parameter and CD45 marker. After capitation of CD7+ T cells, CD56+ NK cells, CD24 + granulocytes and B cells, CD16 + granulocytes, monocyte subclasses were defined by their CD14+ and CD16+ expression. Classical MO: CD14+/CD16-, intermediate MO: CD14+/CD16+, non-classical MO: CD14-/CD16+. Siglec-1 expression levels of the above monocyte subsets were compared to granulocytes.
